# Plasmatic GABA levels and its relation with GRIN2B haplotypes and treatment resistant depression patients.

**DOI:** 10.1192/j.eurpsy.2026.10586

**Published:** 2026-07-31

**Authors:** E. Saez, M. Gomez-Revuelta, O. Olivas, M. I. Macias, M. Unceta, A. Arza, S. Iñiguez, A. Arrue, L. Erkoreka

**Affiliations:** 1 Department of Psychiatry, Galdakao-Usansolo University Hospital, Osakidetza, Galdakao; 2 Mental Health Network Group, Biobizkaia Health Research Institute, Barakaldo; 3 Department of Psychiatry, Marques de Valdecilla University Hospital; 4 IDIVAL Research Institute; 5 Department of Psychiatry, Cantabria University, Santander; 6 Department of Psychiatry, Mental Health Center,Mental Health Network Bizkaia, Osakidetza, Basauri; 7 Occupational Health, Mental Health Network Bizkaia, Osakidetza, Bilbao; 8 Clinical Biochemistry Service, Cruces University Hospital, Osakidetza; 9 Biobizkaia Health Research Institute; 10 Neurochemical Research, Mental Health Network Bizkaia, Osakidetza, Barakaldo; 11 Department of Psychiatry, University of the Basque Country, Leioa, Spain

## Abstract

**Introduction:**

The glutamatergic system is implicated in the pathogenesis of Treatment Resistant Depression (TRD), evidenced by the efficacy of ketamine, a N-methyl-D-aspartate receptor (NMDAR) antagonist. This hypothesis suggests that NMDAR inhibition promotes glutamate release via suppression of NMDAR GABAergic interneurons (1). Studies report reduced plasmatic Gamma AminoButiric Acid (pGABA) (2) and altered plasmatic glutamate (pGlu) levels in depressed patients vs. healthy controls (HC) (3). Single nucleotide polymorphisms (SNPs) in the GRIN2B gene, encoding the NMDAR 2B subunit (rs1806201, rs890, rs1805502) have been associated with TRD (4).

**Objectives:**

1. To evaluate the distribution of GRIN2B haplotypes formed by these SNPs (rs1806201-rs890-rs1805502) in TRD and HC. 2. To analyze the relationship between pGABA or pGlu levels and haplotypes in all participants as well as differences by diagnosis.

**Methods:**

38 TRD patients (non-responders to 3 different antidepressants including potentiation strategies) prior to esketamine initiation and 55 HCs were recruited. pGABA and pGLu levels were analysed by HPLC. Haplotypes were inferred using PLINK toolset. Associations with diagnosis were assessed by PLINK toolset and Fisher’s test. General Linear models (GLM) were used to evaluate haplotype effects on pGABA or pGlu levels in all participants as well as differences by diagnosis, considering age and sex were covariates.

**Results:**

No differences in GRIN2B haplotype distribution between TRD and HC were observed. In GLM analysis, only ACG haplotype (rs1806201-rs890-rs1805502) showed effect on pGABA (Wald’s X^2^ = 11.77, df:5 p = 0.038). While no overall association was found between AGC-haplotype presence and pGABA levels (B = 0.153, p = 0.166), a diagnosis-haplotype interaction was observed (B = -0.110, p = 0.033). Among TRD patients, AGC carriers had higher pGABA levels than non-AGC carriers, whereas no difference was observed in HCs. Moreover, TRD patients had lower pGABA levels than HCs (B = 0.125, p = 0.006) and pGABA levels increase with age in the study population (B = 0.002, p = 0.043). Sex had no effect. In pGlu no relevant findings were observed

**Conclusions:**

While GRIN2B haplotypes seemed to be not associated with TRD diagnosis, the pGABA levels are related to TRD and are influenced by ACG-haplotype in TRD. Our findings suggest a potential role of the AGC-haplotype (rs1806201-rs890-rs1805502) modulating pGABA levels among TRD patients. These results could improve treatment selection in these patients. Nonetheless, given our limited sample size, larger samples are necessary to confirm these results.

**References:**

1. Zanos P, Gould TD. Mol Psychiatry. 2018;23(4):801-11.

2. Gammoh O, et al. XCLI J. 2024 Jan 4;23:62–78.

3. Romeo B, et al. J Psychiatry Neurosci JPN. 2018;43(1):58-66.

4. Zhang C, et al. Psychopharmacology. 2014; 231: 685-693.

Supported by the Basque Government Health Department, 2023111045

**Disclosure of Interest:**

None Declared

